# Effect of a DPP-4 Inhibitor on Orthodontic Tooth Movement and Associated Root Resorption

**DOI:** 10.1155/2020/7189084

**Published:** 2020-08-18

**Authors:** Jiawei Qi, Hideki Kitaura, Wei-Ren Shen, Saika Ogawa, Fumitoshi Ohori, Takahiro Noguchi, Aseel Marahleh, Yasuhiko Nara, Pramusita Adya, Itaru Mizoguchi

**Affiliations:** Division of Orthodontics and Dentofacial Orthopedics, Graduate School of Dentistry, Tohoku University, Sendai, Japan

## Abstract

**Objectives:**

Dipeptidyl peptidase-4 (DPP-4) inhibitors are used as a treatment for type 2 diabetes mellitus and have also recently been applied to enhance bone quality and density, and increase the expression of bone markers. This study aimed to investigate the effect of a DPP-4 inhibitor on orthodontic tooth movement (OTM) and related root resorption in a mouse model.

**Materials and Methods:**

Mice were randomly divided into three groups: those undergoing OTM with the addition of a DPP-4 inhibitor (30 *μ*g), those undergoing OTM and receiving phosphate-buffered saline (PBS), and those without force loading (control group). OTM was achieved by means of a nickel–titanium closed coil spring that moved the first molar in a mesial direction for 12 days. The distance of OTM was measured using silicone impression. Maxillae were removed for histological analysis or real-time PCR analysis.

**Results:**

The distance of OTM and the number of osteoclasts were significantly decreased after administration of the DPP-4 inhibitor, which also significantly suppressed the number of odontoclasts and root resorption after OTM. Furthermore, the mRNA expression of tumour necrosis factor-*α* (TNF-*α*) and the receptor activator of nuclear factor kappa-B ligand (RANKL) were decreased in DPP-4 inhibitor-treated mice compared with those receiving PBS and control animals.

**Conclusion:**

The DPP-4 inhibitor inhibited tooth movement and associated root resorption by blocking the formation of osteoclasts and odontoclasts, respectively. It also appeared to inhibit osteoclastogenesis and odontoclastogenesis by suppressing the expression of TNF-*α* and/or RANKL.

## 1. Introduction

Type 2 diabetes mellitus is a major public health issue, and the number of patients is increasing worldwide. Affected patients have a higher risk of bone fracture than healthy individuals [1]. Dipeptidyl peptidase-4 (DPP-4) inhibitors, an antidiabetic medication, initially inhibit the enzymatic activity of DPP-4. Subsequently, the degradation of incretin hormones that stimulate insulin secretion from pancreatic *β* cells is inhibited, which ultimately controls blood glucose levels [2].

In recent years, the influence of DPP-4 inhibitors on bone metabolism has been widely studied. The effect whether DPP-4 inhibitors can reduce the risk of bone fracture still remained controversial. Some researchers believed that compared with other antidiabetic drugs, DPP-4 inhibitors showed a lower fracture risk in clinical studies [3, 4]. Conversely, Hidayat et al. argued that it was no effects of DPP-4 inhibitors on the risk of fracture according to cumulative real-world evidence [5]. In animal experiments, it was exhibited positive effects on bone metabolism by enhancing bone quality and density, and the expression of bone markers [6]. Additionally, a DPP-4 inhibitor had a protective effect against tumour necrosis factor (TNF)-*α*-induced chondrocyte senescence [7], while we previously showed that a DPP-4 inhibitor inhibited lipopolysaccharide (LPS)-induced osteoclast formation and bone resorption by decreasing LPS-induced TNF-*α* expression in macrophages [8].

Osteoclasts, derived from haematopoietic stem cells, regulate the resorption of bone during its remodelling. Macrophage colony-stimulating factor (M-CSF) and the ligand for the receptor activator of necrosis factor *κ*B (RANKL) are two important cytokines for osteoclast differentiation and formation [9]. TNF-*α* has also been reported to be another essential cytokine for osteoclastogenesis [10–12].

Orthodontic tooth movement (OTM) is achieved by remodelling of the periodontal ligament and alveolar bone upon application of an external force. The mechanism of OTM has proven to be a multifactorial process involving molecules such as neurotransmitters, cytokines, growth factors, and bone matrix constituents. These molecules mediate the differentiation and function of osteoclasts and osteoblasts, leading to bone remodelling [13–17].

In previous studies, TNF-*α* was shown to be induced after mechanical force loading [18]. TNF receptor-deficient mice demonstrated reduced tooth movement compared with wild-type mice, indicating that TNF-*α* plays an essential role in osteoclast formation and bone remodelling during OTM [19, 20]. However, the effect of DPP-4 inhibitors on OTM remains largely unknown. A previous study showed that the rate of orthodontic tooth movement is also closely related to the turnover rate of alveolar bone in rat [21]. Therefore, in the present study, we established a mouse model of OTM to evaluate the effect of a DPP-4 inhibitor on OTM, the level of osteoclast activity, and root resorption.

## 2. Materials and Methods

### 2.1. Ethical Statement

All animal procedures and protocols were performed in accordance with the guidelines of the animal care and use committee of the Tohoku University. The institutional committee on the ethics of animal experiments approved the study protocol (permit number: 2019DnA-047-2).

### 2.2. Experimental Animals and Reagents

C57BL6/J male mice (8–10 weeks old) were obtained from CLEA Japan Inc. (Tokyo, Japan) and housed in cages in a room maintained at 21–24°C with a 12 h/12 h light/dark cycle. 24 mice were totally used in this study. The mice were fed a granular diet (Oriental Yeast, Tokyo, Japan) to prevent eating difficulties during force-loading. The DPP-4 inhibitor linagliptin was purchased from R&D Systems (Minneapolis, MN, USA).

### 2.3. Orthodontic Tooth Movement

**Figure 1:**
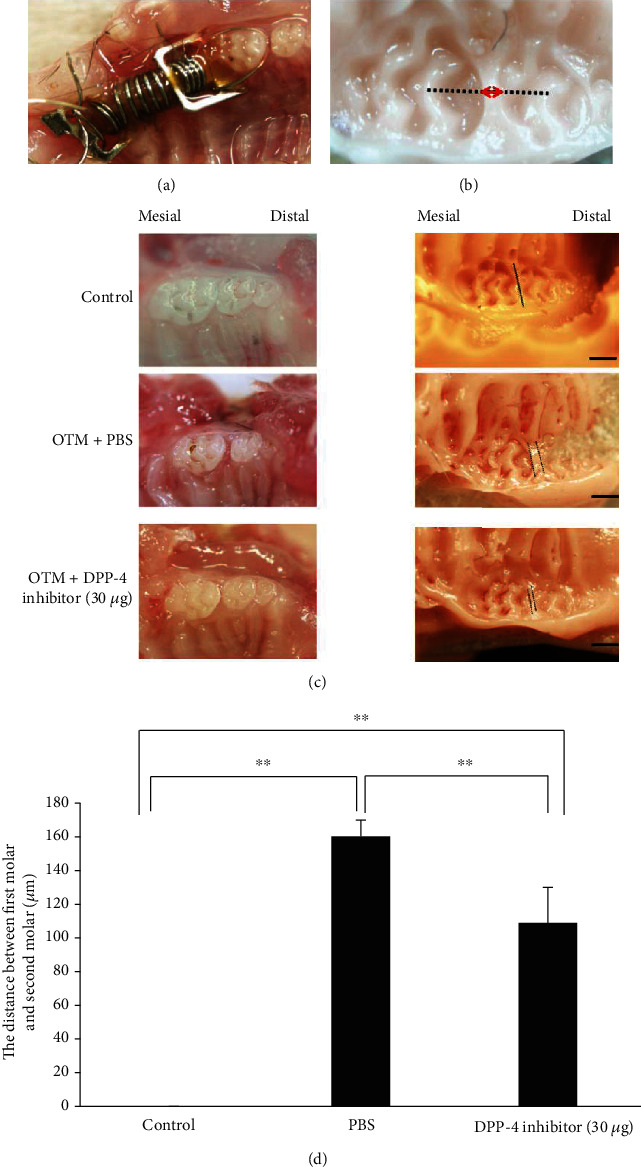
Orthodontic appliance and effect of DPP-4 inhibitor on orthodontic tooth movement. (a) Intraoral photograph of the appliance fixed between incisor and first molar. (b) Photograph of the silicone impression with stereoscopic microscope after tooth movement. The dashed line connecting the central fossae of the first and second molars was used to measure the distance from the distal marginal ridge of the first molar to the mesial marginal ridge of the second molar. (red double arrow). (c) Intraoral photographs of the upper left molars after 12 days of tooth movement with administration of PBS or 30 *μ*g of DPP-4 inhibitor, and the control (unloaded). Tooth movement distances were measured by taking silicone impressions. Scale bars = 500 *μ*m. (d) Comparison of tooth movement among the three groups. *n* = 4 for each group. ^∗∗^*P* < 0.01.

Mice were anaesthetized on each experimental time point. A combination anesthetic including medetomidine, midazolam, and butorphanol was intraperitoneally injected into mice. An orthodontic appliance was used to move the first molar in a mesial direction, as described previously [22]. Briefly, a nickel–titanium closed coil spring (Tomy; Fukushima, Japan) was fixed between the upper incisors and the upper-left first molar of mice with a 0.1-mm stainless steel wire (Figure 1(a)). According to the manufacturer, OTM was achieved after force-loading for 12 days using a force of approximately 10 g after activation. The method of injection was the same as mentioned previously [19]. Linagliptin was dissolved in phosphate-buffered saline (PBS; 30 *μ*l). Mice were injected every 2 days for a total of 7 injections under anaesthesia. Injections were directed into the buccal gingiva close to the space between upper-left first and second molars during OTM using a 0.5-ml syringe with a 30 G 10-mm needle (Nipro, Osaka, Japan). Only one injection site was at each time. The depth of injection was approximately from gingiva surface to bone surface. Mice were randomly divided into three groups: those receiving OTM with linagliptin (30 *μ*g) every 2 days, those receiving OTM with PBS every 2 days, and those without force loading (control group).

### 2.4. Measurement of Tooth Movement

The mice were anaesthetised after 12 days of OTM. We measured the space between the first and second molars using a tray containing hydrophilic vinylpolysiloxane (EXAFAST Injection Type, GC Co., Tokyo, Japan) to obtain an impression of the maxillary teeth. We used stereoscopic microscopy (VH-7000; Keyence, Osaka, Japan) to evaluate tooth movement with the closest distance between the distal marginal ridge of the first molar and the mesial marginal ridge of the second molar (Figure 1(b)).

### 2.5. Preparation of RNA and Real-Time PCR Analysis

For our *in vivo* experiment, maxillae were removed and the left side of the maxilla around the upper first molar was placed in liquid nitrogen, then centrifuged in 800 *μ*l TRIzol reagent (Invitrogen, Carlsbad, CA, USA) by a Micro Smash MS-100R homogenising system (Tomy Seiko, Tokyo, Japan). RNA extraction was performed using a RNeasy mini kit (Qiagen, Valencia, CA, USA) according to the manufacturer's instructions. Total RNA was isolated from left side maxilla. cDNA was synthesised using 2 *μ*g of total RNA with oligo-dT primers (Invitrogen) and reverse transcriptase in a volume of 20 *μ*l. The relative expression of RANKL, TNF-*α*, and osteoprotegerin (OPG) mRNA was normalised to glyceraldehyde 3-phosphate dehydrogenase (GAPDH) mRNA and measured by real-time PCR in a Thermal Cycler Dice Real Time system (Takara, Shiga, Japan). Each well contained 2 *μ*l cDNA, a 23 *μ*l mixture of SYBR Premix Ex Taq (Takara), and 50 pmol/*μ*l primers. Cycling conditions were the following: initial denaturation at 95°C for 30 s, then 50 cycles of 95°C for 5 s, 60°C for 30 s, and a final dissociation stage (95°C for 15 s, 60°C for 30 s, and 95°C for 15 s). Primers were as follows: GAPDH, 5′-GGTGGAGCCAAAAGGGTCA-3′ and 5′-GGGGGCTAAGCAGTTGGT-3′; RANKL, 5′-CCTGAGGCCAGCCATTT-3′ and 5′-CTTGGCCCAGCCTCGAT-3′; TNF-*α*, 5′-CTGTAGCCCACGTCGTAGC-3′ and 5′-TTGAGATCCATGCCGTTG-3′; OPG, 5′-ATCAGAGCCTCATCACCTT-3′ and 5′-CTTAGGTCCAACTACAGAGGAAC-3′ [8].

### 2.6. Histological Analysis

After OTM for 12 days, the maxillae were obtained and fixed in 4% paraformaldehyde overnight at room temperature. The tissue was decalcified in 14% ethylene diamine tetra-acetate for 3 weeks at room temperature, then paraffin-embedded, and sectioned in the horizontal plane at 4 *μ*m for histological analysis. The distobuccal root of the first molar was evaluated in each sample, and five levels in each sample were assessed: 100, 140, 180, 220, and 260 *μ*m away from the bifurcation surface. After deparaffinisation, the sections were stained for tartrate-resistant acid phosphatase (TRAP) activity and counterstained with haematoxylin. The TRAP staining solution consisted of naphthol-ASMX-phosphate (Sigma-Aldrich; St Louis, Missouri, USA), Fast Red Violet LB Salt (Sigma-Aldrich), and 50 mM sodium tartrate. Under light microscopy, osteoclasts were considered as TRAP-positive multinuclear cells, located in lacunae in the resorbed alveolar bone surface. Conversely, odontoclasts were considered as TRAP-positive multinuclear cells located in lacunae in the resorbed root surface. The number of TRAP-positive cells was evaluated on the mesial side of the distobuccal root of the upper-left first molar. The mean values were calculated in all the five sections. The ratio of the root resorption area was calculated by the percentage of resorption surface/root surface. The surface area was measured using ImageJ software (National Institutes of Health, Bethesda, Maryland, USA [23].

### 2.7. Statistical Analysis

All data values were presented as the mean ± standard deviation (SD) and were assessed by Scheffe's *F*-tests and Student's *t*-tests. Differences with *P* < 0.05 were considered statistically significant.

## 3. Results

### 3.1. Effect of the DPP-4 Inhibitor on Orthodontic Tooth Movement

No significant space between the first and second molars was observed in the control group (without force loading). Tooth movement in the mesial direction was observed for both experimental groups after force loading for 12 days. The mean distance between the upper-left first molar and second molar was 160.31 ± 9.73 *μ*m in the PBS injection group, but this was significantly reduced to 108.90 ± 21.20 *μ*m in the group treated with the DPP-4 inhibitor. This indicates that OTM was inhibited by the local administration of a DPP-4 inhibitor (Figures 1(c) and 1(d)).

### 3.2. Effect of the DPP-4 Inhibitor on the Number of TRAP-Positive Osteoclasts along the Alveolar Bone

**Figure 2:**
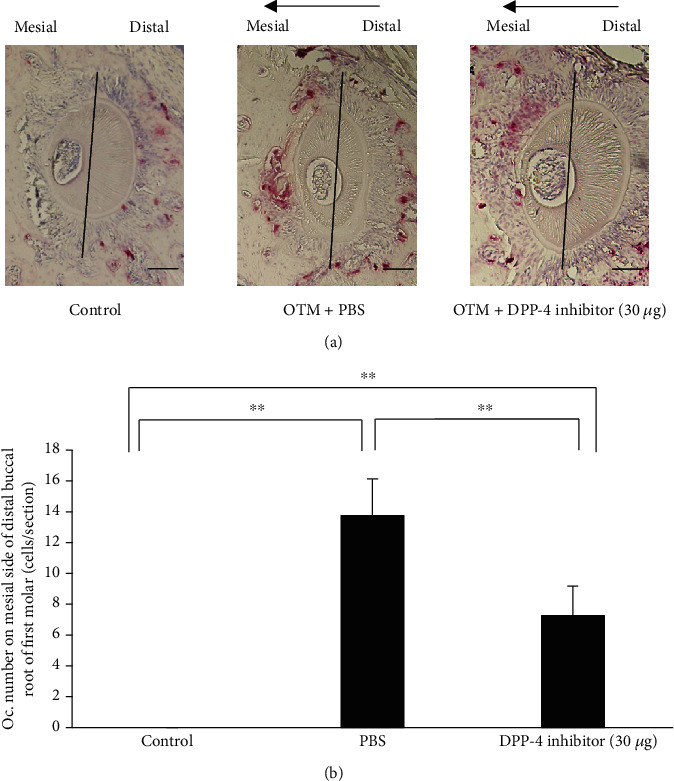
Histology analysis of mouse alveolar bone in the maxillary left first molar area in horizontal sections. (a) TRAP-stained histological sections of the distobuccal root of the maxillary left first molar before and after 12 days of experimental tooth movement with administration of PBS or 30 *μ*g of DPP-4 inhibitor. Arrows represent the direction of orthodontic tooth movement. (b) Evaluation of the number of TRAP-positive multinucleated cells on the mesial side of the distobuccal upper-left first molar. *n* = 4 for each group. ^∗∗^*P* < 0.01.

TRAP staining was performed on tissue sections from the distobuccal root of the upper-left first molar in control and experimental groups. In the control group, no TRAP-positive osteoclasts were detected along the alveolar bone on the mesial side of the root. However, force loading for 12 days in the experimental groups significantly increased the osteoclast number compared with the control group. Furthermore, mice injected with the DPP-4 inhibitor demonstrated a significantly reduced number of TRAP-positive osteoclasts (7.25 ± 1.92 cells/section) compared with PBS-administered mice (13.75 ± 2.38 cells/section) (Figures 2(a) and 2(b)).

### 3.3. Effect of the DPP-4 Inhibitor on Mechanical Force-Induced Root Resorption

**Figure 3:**
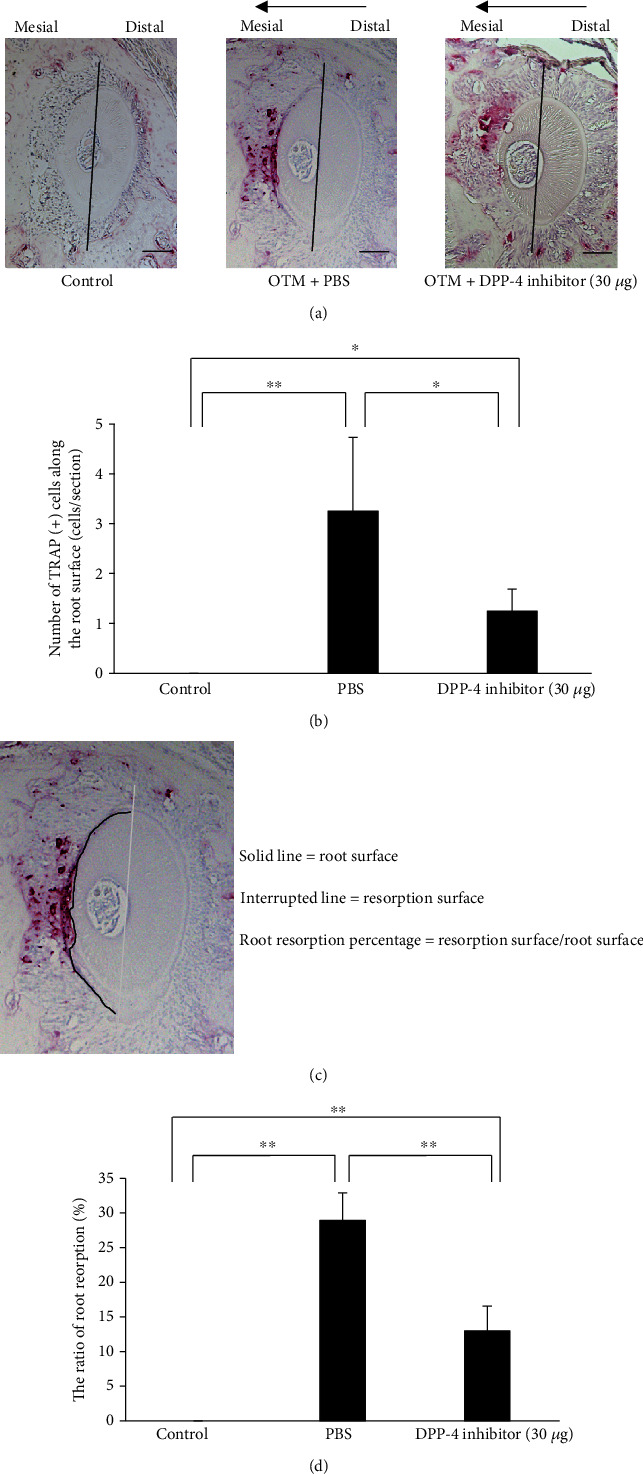
Evaluation of odontoclast activity and root resorption on the transverse histological sections. (a) TRAP-stained histological sections of the distobuccal root of the maxillary left first molar before and after 12 days of experimental tooth movement with administration of PBS or 30 *μ*g of DPP-4 inhibitor. Arrows represent the direction of orthodontic tooth movement. (b) The number of TRAP-positive multinuclear cells in mice along the root surface on the mesial side. *n* = 4 for each group. ^∗^*P* < 0.05; ^∗∗^*P* < 0.01. (c) The evaluation of the root resorption surface with histological sections. Solid line indicates the root surface, and the interrupted line indicates the resorption surface. The root resorption surface was measured by the percentage of interrupted line/solid line. Scale bars = 100 *μ*m. (d) The ratio of the root resorption surface in control group and experimental groups treated with PBS or DPP-4 inhibitor. *n* = 4 for each group. ^∗^*P* < 0.05; ^∗∗^*P* < 0.01.

Next, the odontoclast number was evaluated on the root surface of the mesial side of the distobuccal root after 12 days of tooth movement. Odontoclasts were significantly increased in number in PBS-administered mice (3.25 ± 1.48 cells/section) compared with controls, but significantly decreased to 1.25 ± 0.43 cells/section after treatment with the DPP-4 inhibitor (Figures 3(a) and 3(b)). The root resorption area was also assessed using a stereoscopic microscope. Transverse paraffin sections showed an increase of the surface area of root resorption in PBS-administered mice compared with the control group. The area of root resorption was significantly smaller in DPP-4 inhibitor-administered mice than in those that received PBS (28.95 ± 3.97 and 12.98 ± 3.58%, respectively), but greater than in the control group. This indicated that odontoclast activity and root resorption were partially inhibited by the local injection of a DPP-4 inhibitor (Figures 3(c) and 3(d)).

### 3.4. Effect of the DPP-4 Inhibitor on the Expression of RANKL, TNF-*α*, and OPG *in vivo*

**Figure 4:**
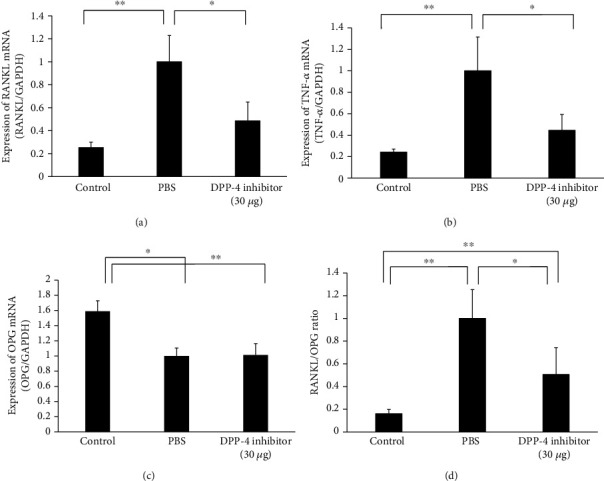
The effect of DPP-4 inhibitor on expressions of RANKL, TNF-*α*, OPG, and RANKL/OPG ratio *in vivo.* (a–d) Relative expression levels of RANKL, TNF-*α*, and OPG mRNA in mouse alveolar bone detected by real-time PCR. RANKL, TNF-*α*, and OPG mRNA levels were normalized to the levels of GAPDH. *n* = 4 for each group. ^∗^*P* < 0.05; ^∗∗^*P* < 0.01.

Alveolar bone surrounding the first molar was isolated after 12 days of tooth movement, and the expression of RANKL, TNF-*α*, and OPG mRNA was measured with real-time PCR. The PBS-administered group showed a significant increase in RANKL, TNF-*α* mRNA, and RANKL/OPG ratio compared with the control group, while the DPP-4 inhibitor-administered group showed a significant decrease in RANKL, TNF-*α* mRNA levels, and RANKL/OPG ratio compared with the PBS-administered group. OPG expression was decreased after OTM. However, it was showed no difference in the expression of OPG mRNA between PBS and DPP-4 inhibitor injection group. (Figures 4(a)–4(d)).

## 4. Discussion

Type 2 diabetes mellitus is a metabolic disorder with reduction in metabolic and immune [24], and the number of affected patients is showing an increasing global trend [25]. It has recently been identified as an important risk factor for osteoporosis-associated fractures [26, 27], but the long-term use of some antidiabetic drugs was reported to have side effects for bone metabolism [28, 29]. Indeed, a DPP-4 inhibitor was recently shown to have anti-inflammatory actions in several types of vascular cells and immune cells [30, 31], while linagliptin has potent beneficial effects in some inflammatory diseases [8, 32]. There are few reports regarding the outcome of short-term DPP-4 inhibitor administration, and this study is the first to report the effect of DPP-4 inhibition on OTM and associated root resorption.

We initially investigated the effects of linagliptin on mechanical tooth movement in mice. We observed tooth movement of 160.31 ± 9.73 *μ*m after 12 days; this is similar to that reported in our previous studies, suggesting that the OTM mouse is a reliable animal model. We previously administered 30 *μ*g (nearly 1.5 mg/kg/day) linagliptin as a DPP-4 inhibitor [8] to inhibit LPS-induced inflammation in mouse calvaria, so used the same concentration of linagliptin in the present study. OTM in mice is a multifactorial process affected by the type of appliance, magnitude and direction of the force, and type of tooth movement [33]. In the present study, we found that the local administration of linagliptin reduced the distance of tooth movement compared with the PBS-administered group.

Bone remodelling plays an important role in the mechanism of tooth movement. Osteoclast activation on the pressure side is responsible for mechanical stress in tooth movement. Therefore, we analysed osteoclast formation in histological sections of the distal buccal root of the upper-left first molar. We found that the osteoclast number on the mesial side was significantly decreased in the DPP-4 inhibitor group compared with the PBS group, indicating that the suppressed tooth displacement rate following local injection of a DPP4 inhibitor may be via a reduction in osteoclast formation.

Root resorption is an unavoidable iatrogenic outcome after orthodontic treatment [34]. In this study, an obvious root resorbed area along the mesial side of the distobuccal root was observed in PBS-injected mice after 12 days of tooth movement. However, this area of root resorption was significantly reduced in the DPP4 inhibitor-injected group. Odontoclasts are responsible for root resorption, and their number was significantly decreased on the mesial side of the distobuccal root in mice treated with the DPP-4 inhibitor compared with PBS-treated mice. However, odontoclast number and root resorption ratio were still significantly higher than in control mice, suggesting that only partial inhibition of root resorption was achieved by the DPP-4 inhibitor and that this was likely to be via inhibition of odontoclast activity.

Increased RANKL production with osteoclast-induced tooth movement was previously demonstrated in osteoprotegerin-deficient mice, indicating that RANKL plays a critical role in osteoclast differentiation during OTM [35]. TNF-*α* was also reported to enhance osteoclast-induced bone resorption during OTM in TNF receptor-deficient mice [19]. It reported that OPG can downregulate osteoclast formation and orthodontic tooth movement [36]. The balance of RANKL/OPG also plays an important role in orthodontic tooth movement [37]. However, it was unclear how DPP-4 inhibitors suppress osteoclast activity. The expression of OPG was not significantly affected by DPP-4 inhibitor in this study. Our current analysis showed that RANKL and TNF-*α* mRNA expression in alveolar bone was inhibited in mice receiving the DPP-4 inhibitor compared with PBS-administered mice. Therefore, RANKL/OPG ratio was decreased in DPP-4 inhibitor-administered group. We previously found that TNF-*α* plays an important role in sclerostin-induced RANKL expression during OTM [38]. Although the expression of RANKL in alveolar bone was suppressed after the injection of linagliptin, it is not known whether linagliptin directly inhibits RANKL expression during OTM, so further studies are required to evaluate this.

Macrophages are classified as M1 (classically activated macrophages) and M2 (alternatively activated macrophages). M1 macrophages have a proinflammatory function involving the secretion of high levels of nitric oxide and proinflammatory cytokines such as TNF-*α* [39]. Conversely, M2 macrophages secrete anti-inflammatory interleukin-10 and arginase-1 [40, 41]. A decrease in the M1/M2 macrophage ratio was shown to inhibit alveolar bone resorption in mouse periodontitis models [42], while root resorption promoted by an increased M1/M2 macrophage ratio and high levels of TNF-*α* was observed in rats after 7 days of OTM [43]. He et al. found that M1, but not M2, macrophages were significantly decreased after DPP-4 inhibitor treatments, resulting in a significant decrease in the M1/M2 macrophage ratio [44]. Similarly, we previously showed that a DPP-4 inhibitor suppressed LPS-induced TNF-*α* expression in mouse calvaria [8]. Taking these findings together with the present results, it appears that TNF-*α*-induced osteoclast formation and root resorption may be inhibited by downregulation of the M1/M2 macrophage ratio in response to linagliptin treatment.

## 5. Conclusion

The present findings demonstrate that treatment with a DPP-4 inhibitor inhibits tooth movement and associated root resorption by inhibiting the formation of osteoclasts and odontoclasts, respectively. Additionally, DPP-4 inhibitors may inhibit osteoclastogenesis and odontoclastogenesis by suppressing TNF-*α* and/or RANKL expression. Based on these findings, we propose that more attention should be paid to orthodontic patients receiving DPP-4 inhibitors for diabetes treatment.

## Data Availability

The data used in the present study to support the findings are available from the corresponding author on reasonable request.
